# Panspecies molecular assays detect viral pathogens missed by real-time PCR/reverse-transcriptase PCR among pneumonia patients, Sarawak, Malaysia

**DOI:** 10.1186/s40794-020-00114-2

**Published:** 2020-08-12

**Authors:** Jane K. Fieldhouse, Emily S. Bailey, Teck-Hock Toh, King-Ching Hii, Kerry A. Mallinson, Jakie Ting, John A. Lednicky, Antoinette Berita, Tham Thi Nguyen, Diego Galan, Son T. Than, See-Chang Wong, Toh-Mee Wong, Patrick J. Blair, Gregory C. Gray

**Affiliations:** 1 Division of Infectious Diseases, Duke University School of Medicine, DUMC Box 102359, Durham, NC 27710 USA; 2 Duke Global Health Institute, Duke University, Durham, North Carolina USA; 3 Institute for Global Health Sciences, University of California, San Francisco, California USA; 4 Department of Public Health, Texas Tech University Health Sciences Center, Abilene, TX USA; 5 Clinical Research Center, Sibu Hospital, Ministry of Health Malaysia, Sibu, Sarawak Malaysia; 6 Faculty of Medicine, SEGi University, Kota Damansara, Selangor Malaysia; 7 Department of Paediatrics, Sibu Hospital, Ministry of Health Malaysia, Sibu, Sarawak Malaysia; 8 Kapit Hospital, Ministry of Health Malaysia, Kapit, Sarawak Malaysia; 9 Department of Environmental and Global Health, University of Florida, Gainesville, Florida USA; 10 Emerging Pathogens Institute, University of Florida, Gainesville, Florida USA; 11 Emerging Infectious Disease Program, Duke-NUS Medical School, Singapore, Singapore; 12 Department of Medicine, Sibu Hospital, Ministry of Health Malaysia, Sibu, Sarawak Malaysia; 13 Naval Medical Research Center-Asia, Singapore, Singapore; 14 Global Health Center, Duke Kunshan University, Kunshan, China

**Keywords:** Pneumonia, Respiratory viruses, Molecular assay, Panspecies, Malaysia

## Abstract

**Background:**

In a year-long pneumonia etiology study conducted June 2017 to May 2018 in Sarawak, Malaysia, 599 patients’ nasopharyngeal swab specimens were studied with real-time polymerase chain reaction (rPCR)/ reverse-transcription (rRT-PCR) assays for respiratory pathogens known to contribute to the high burden of lower respiratory tract infections. The study team sought to compare real-time assay results with panspecies conventional molecular diagnostics to compare sensitivities and learn if novel viruses had been missed.

**Methods:**

Specimens were studied for evidence of adenovirus (AdV), enterovirus (EV) and coronavirus (CoV) with panspecies gel-based nested PCR/RT-PCR assays. Gene sequences of specimens positive by panspecies assays were sequenced and studied with the NCBI Basic Local Alignment Search Tool software.

**Results:**

There was considerable discordance between real-time and conventional molecular methods. The real-time AdV assay found a positivity of 10.4%; however, the AdV panspecies assay detected a positivity of 12.4% and the conventional AdV-Hexon assay detected a positivity of 19.6%. The CoV and EV panspecies assays similarly detected more positive specimens than the real-time assays, with a positivity of 7.8% by the CoV panspecies assay versus 4.2% by rRT-PCR, and 8.0% by the EV panspecies assay versus 1.0% by rRT-PCR. We were not able to ascertain virus viability in this setting. While most discordance was likely due to assay sensitivity for previously described human viruses, two novel, possible zoonotic AdV were detected.

**Conclusions:**

The observed differences in the two modes of amplification suggest that where a problem with sensitivity is suspected, real-time assay results might be supplemented with panspecies conventional PCR/RT-PCR assays.

## Background

Despite the availability of antibiotic and antiviral therapies, advancements in vaccinations, and increased access to health care, the worldwide burden of lower respiratory tract infections (LRTI) remains immense [1], with an estimated 2.38 million deaths (all ages) attributable to LRTIs in 2016 alone [2]. Among the respiratory pathogens contributing to the high burden of LRTI morbidity and mortality are several viruses with known or suspected zoonotic transmission potential [3]. In addition to influenza viruses, these include coronaviruses (CoV), enteroviruses (EV) [4–6] and adenoviruses [7–10]. A systematic review of the viral etiology of community-acquired pneumonia (CAP) published in 2016 found that the highest proportion of viral infection was by influenza virus, detected in 8% of cases, followed by infection by human rhinovirus (HRV), detected in 5.7% of cases. AdV and CoV were detected in 1–4% of CAP patients and EV were detected in < 1% [11].

Clinical diagnostics used to detect CoV, EV, and AdV are often designed using the gene sequences of previously identified viruses which have infected humans. Such diagnostics may miss a novel virus strain, especially such viruses which are naturally harbored in animals. Sarawak, rich in flora and fauna biodiversity [12], is located near the equator with a population that often has considerable exposure to both domestic animals and wildlife. Hence, we hypothesized that in our assessments of the etiology of pneumonia in a study population residing in an area of great animal and plant biodiversity we might be missing animal CoV, EV, and AdV causing human disease. In the following analyses we sought to compare real-time assay results with panspecies conventional molecular diagnostics to examine sensitivities and learn if novel viruses had been missed.

## Methods

In this work we further analyzed 599 nasopharyngeal swab specimens collected during a year-long pneumonia etiology study, conducted between June 2017 and May 2018 in Sarawak, Malaysia [13]. All specimens, regardless of their real-time polymerase chain reaction (rPCR) or real-time reverse transcription polymerase chain reaction (rRT-PCR) assays results, were re-examined for evidence of AdV, EV, and CoV with panspecies gel-based nested PCR/RT-PCR assays. Gene sequences of all specimens found positive by the panspecies assays were then sent for sequencing and studied with the Basic Local Alignment Search Tool software (BLAST) at the National Center for Biotechnology Information (NCBI), recording all matches with greater than 80% identity. Real-time and conventional molecular assays results were then compared.

### Participants

As previously described [13], study enrollment took place from June 15, 2017 to May 14, 2018 at Sibu and Kapit Hospitals in Sarawak, Malaysia. The study team relied on convenience sampling to enroll patients for the first two months of the study before enrolling patients on two out of three randomly selected days of each week, as communicated to medical officers by a study coordinator. Patients of all ages above 30 days who had been admitted to either hospital and diagnosed with pneumonia by an attending physician were considered for study eligibility. Physicians evaluated subjects for inclusion and exclusion criteria, adapted from two large and comprehensive, United States community-based pneumonia studies published in 2015 [14], and confirmed diagnosis by chest radiography within 72 h of hospitalization (see Supplementary Table 1). Adults 18 years of age or older provided written consent and children ages 7 to 18 provided written assent and written parental or guardian consent. For patients < 7 years of age only parental or guardian consent was required. The study was conducted in accordance with the ethical standards of the Helsinki Declaration and received a scientific review by and ethical approval from the Malaysian Ministry of Health’s Medical Research and Ethics Committee, the Duke University Health System Institutional Review Board, the Duke-NUS Medical School Ethical Review Board, and the Naval Medical Research Center-Asia Human Research Protection Program.

### Sampling procedures

Consenting or assenting patients completed a brief questionnaire then permitted the collection of one nasopharyngeal (NP) swab, which was placed into a transport tube with 3 mL sterile viral transport medium (BD Universal Viral Transport; Becton, Dickinson and Company, Franklin Lakes, NJ) and delivered to the Sibu Hospital Clinical Research Center (SHCRC) laboratory or the Kapit Hospital laboratory where the specimen was stored at − 80 °C until RNA or DNA extraction was performed using the QIAmp Cador Pathogen Mini Kit (Qiagen, Hilden, Germany).

### Real-time molecular assays for known human respiratory pathogens

As previously reported [13], rPCR and rRT-PCR assays were initially conducted on similar BioRad CFx96 C1000 Touch Thermal Cycler Real-Time systems at SHCRC. All assays were conducted using DNA or cDNA positive controls and a nuclease-free water negative control. Cycle threshold (Ct) values < 38 were considered positive and Ct values > 40 were considered negative. Ct values 38 to 40 were considered suspect, acknowledging that positivity may be the result of cross-reactivity or nonspecific amplification.

One milliliter aliquots of all specimens were shipped on dry ice then further validated at the Duke One Health Research Laboratory at Duke University in Durham, North Carolina (*n* = 428) or the Laboratory of One Health Research at Duke-NUS Medical School in Singapore (*n* = 171) where real-time assays were repeated and results were agreed upon [13].

### Conventional molecular assays for human or animal respiratory pathogens

Specimens which had not been depleted from previous work were examined with new nucleic extractions and panspecies conventional PCR/RT-PCR assays at either Duke University or Duke-NUS Medical School. These panspecies assays were designed to detect both human and animal pathogens.

Viral DNA was assessed for AdV using a gel-based nested PCR assay with Invitrogen Platinum Taq DNA Polymerase Kit (Thermo Fisher Scientific, Inc., Waltham, MA) as described by Wellehan et al. (Table 1) [15]. Viral RNA was analyzed with gel-based RT-PCR assays for pan-species EV and pan-species CoV using SuperScript® III One-Step RT-PCR System with Platinum® Taq DNA Polymerase as described by Vijgen et al. and the World Health Organization [17, 18]. Specimens positive by the pan-species assays at Duke University and Duke-NUS University were sent for sequencing at Eton Bioscience, Inc., in Durham, North Carolina, and AITbiotech in Singapore, respectively. CoV positives were sequenced using the RT-PCR primers described in Lelli et al., 2013 [16].

**Table 1:** Primer and probe sequences and gene target region for all panspecies assays. All assays were adapted from the cited references at Duke University

***Virus Assay***	***Function***	***Sequence***	***Target Gene***
Pan adenovirus [15]			
Nested PCR 1	Forward primer (polFouter)	5′-TNMGNGGNGGNMGNTGYTAYCC-3′	Polymerase
	Reverse primer (polRouter)	5′-GTDGCRAANSHNCCRTABARNGMRTT-3′	
Nested PCR 2	Forward primer (polFinner)	5′-GTNTWYGAYATHTGYGGHATGTAYGC-3′	
	Reverse primer (polRinner)	5′-CCANCCBCDRTTRTGNARNGTRA-3′	
Pan coronavirus [16]	Forward primer	5′-GGTTGGGACTATCCTAAGTGTGA-3’	Polymerase
	Reverse primer	5′-CCATCATCAGATAGAATCATCATA-3’	
Pan enterovirus [17]	AN32 primer	5′ GTY TGC CA 3′	Capsid protein VP1
	AN33 primer	5′ GAY TGC CA 3′	
	AN34 primer	5′ CCR TCR TA 3′	
	AN35 primer	5′ RCT YTG CCA 3′	

Select gene sequences were then studied with the NCBI software tool BLAST using the FASTA formatted sequences and the BioEdit program (Ibis Biosciences, Carlsbad, CA, USA). Matches with > 80% identity were reported.

The 428 specimens shipped to Duke University were additionally screened using a gel-based nested PCR assay for human AdV targeting a partial region of the hexon gene using Invitrogen Platinum Taq DNA Polymerase Kit (Thermo Fisher Scientific, Inc., Waltham, MA) [19]. We were unable to conduct the hexon assay on the specimens shipped to Duke-NUS.

### Statistical analysis

Real-time assay results and baseline data from enrollment questionnaires were entered into REDCap version 7.0 before being imported into STATA version 15.0 (StataCorp, College Station, TX) to assess patient characteristics and laboratory results. Comparison of real-time and conventional assay results were conducted using STATA (Cohen’s kappa coefficient) and Microsoft Excel.

## Results

A total of 600 hospitalized pneumonia patients were enrolled at Sibu and Kapit Hospitals between June 15, 2017 and May 15, 2018, with 65% of patients enrolled at Sibu Hospital. Of the enrolled subjects, 325 (54.2%) were male. A total of 385 (64.2%) enrolled subjects were children 5 years of age or younger and 439 (73.2%) were of age 18 years and younger (see Supplementary Table 2).

Of the 600 NP swabs collected, 599 (one NP was accidentally destroyed before molecular screening) were analyzed using rPCR or rRT-PCR for influenza A, B, C, and D viruses, AdV, EV, CoV, respiratory syncytial virus subtype A (RSV-A) or RSV-B, and parainfluenza virus (types 1–4). Four hundred twenty-seven (427) of the total 428 samples at Duke University (one sample was depleted) were additionally analyzed by conventional PCR for HRV species A, B, and C (Table 2) [20] (see Supplementary Tables 3, 4, 5).

**Table 2:** Comparison of adenovirus (AdV), enterovirus (EV), and coronavirus (CoV) assay results across Duke and Duke-NUS analysis

**AdV assays**	**Positive specimens (n)**	**Positivity (%)**
AdV rPCR (*n* = 599)	62	10.4%
AdV panspecies (*n* = 599)	74	12.4%
AdV Hex conventional (*n* = 428)	84	19.6%
**EV assays**	**Positive specimens (n)**	**Positivity (%)**
EV rRT-PCR (*n* = 599)	25	4.2%
EV panspecies (*n* = 599)	48	8.0%
HRV gel electrophoresis & sequence analysis (*n* = 427)	51	11.9%
**CoV assays**	**Positive specimens (n)**	**Positivity (%)**
CoV rRT-PCR (*n* = 599)	6	1.0%
CoV panspecies (*n* = 599)	47	7.8%

### Real-time molecular assays

A total of 62 specimens were positive by rPCR for AdV, with an overall positivity of 10.4%. A total of 25 specimens were positive by rRT-PCR for EV (4.2% positivity) and six specimens were positive by rRT-PCR for CoV (1.0% positivity).

### Conventional assays

We were surprised by the discordance between real-time and conventional molecular methods (Table 2). A total 74 specimens were positive by the panspecies AdV assay (59 at Duke University and 15 at Duke-NUS). Of the 428 specimens analyzed with the human AdV-Hexon assay, 84 specimens were positive, of which, 45 were neither positive by rPCR nor the panspecies assay. There were 44 specimens that were positive by the panspecies AdV assay that were negative by rPCR and a total 32 specimens that were positive by rPCR that were negative by the AdV panspecies assay (see Supplementary Table 6).

Forty-eight (48) of the 599 specimens across Duke (*n* = 24) and Duke-NUS (*n* = 24) were positive by panspecies enterovirus assays, nearly doubling the positivity detected by the rRT-PCR assay (see Supplementary Table 7). Of the subset of 427 specimens analyzed for HRV at Duke University, 51 (11.9%) of the 427 specimens were positive by gel electrophoresis and sequence analysis for HRV. The positivity of the panspecies CoV assay was again much higher than the 1% positivity by the real-time assay, with 47 specimens positive by the panspecies CoV assay, 45 at Duke University and two at Duke-NUS (see Supplementary Table 8).

### Sequencing results

We successfully sequenced 30 of the 74 specimens positive by the panspecies AdV assay. Of the 30 sequenced specimens, 83% were collected from pediatric patients (Table 3). The most prevalent AdV detected was human AdV type 7 (HAdV-7), with 14 total specimens having between 93 and 99% identity with HAdV-7. All 14 of the HAdV-7 specimens were collected from pediatric patients hospitalized at Sibu Hospital (Table 4). In addition to HAdV-7, sequencing revealed several human AdV type 5 (HAdV-5) detections in addition to human AdV type 4 (HAdV-41) and several AdV from the mastadenovirus genera, including the type species human mastadenovirus C (Table 3).

**Table 3:** Results of successfully sequenced specimens positive by panspecies adenovirus [15] assay

	Patient age	Hospital	Sequencing Result	Identity Score From BLAST	Accession Number from BLAST	rPCR Assay	AdV-Hexon Assay
1.	6 months	Kapit	Human mastadenovirus C	98%	MH121117.1	Neg	Neg
2.	65 years	Kapit	Human mastadenovirus E	99%	KY996453.1	Pos	Pos
3.	1 month	Kapit	Human mastadenovirus C	98%	MH121117.1	Neg	Neg
4.	66 years	Sibu	Human mastadenovirus C	97%	MH121097.1	Neg	Neg
5.	1 year	Sibu	Human adenovirus C	95%	MF358574.1	Pos	N/A^a^
6.	10 months	Kapit	Human adenovirus 41	98%	KX868523.2	Neg	N/A^a^
7.	6 months	Sibu	Human adenovirus C	98%	MF358562.1	Pos	N/A^a^
8.	4 months	Kapit	human adenovirus 41	98%	KX868523.2	Neg	N/A^a^
9.	1 year	Kapit	Human adenovirus 5	98%	MF358601.1	Pos	Pos
10.	35 years	Sibu	Human adenovirus 4	99%	AP014851.1	Pos	Pos
11.	3 years	Sibu	Human adenovirus B	99%	KF268212.1	Pos	Neg
12.	2 years	Sibu	Human adenovirus 7	99%	MG923582.1	Pos	Neg
13.	2 years	Sibu	Human adenovirus 7	98%	MG923582.1	Pos	Neg
14.	9 months	Sibu	Human adenovirus 7	98%	MG923582.1	Pos	Neg
15.	6 months	Kapit	Human adenovirus 7	97%	MG923582.1	Pos	Pos
16.	2 years	Sibu	Human adenovirus 7	99%	MG923582.1	Pos	Neg
17.	1 month	Sibu	Human adenovirus 5	99%	MF358604.1	Pos	Neg
18.	8 months	Sibu	Human adenovirus 7	98%	MG923582.1	Pos	Pos
19.	8 months	Sibu	Human adenovirus 7	98%	MG923582.1	Pos	Pos
20.	7 months	Sibu	Human adenovirus 7	98%	MG923582.1	Pos	Pos
21.	1 year	Sibu	Human adenovirus 7	97%	MG923582.1	Pos	Pos
22.	8 months	Sibu	Desmondus rotundus adenovirus 1	100%	KX774303.1	Pos	Pos
23.	1 month	Sibu	Desmondus rotundus adenovirus 1	100%	KX774303.1	Pos	Pos
24.	6 months	Sibu	Human adenovirus 7	96%	MG923582.1	Pos	Pos
25.	3 months	Sibu	Human adenovirus 7	93%	MG923582.1	Pos	Pos
26.	4 months	Sibu	Human adenovirus 7	98%	MG923582.1	Pos	Pos
27.	2 years	Sibu	Human adenovirus 7	98%	MG923582.1	Pos	Pos
28.	2 months	Sibu	Human adenovirus 7	97%	MG923582.1	Pos	Pos
29.	54 years	Sibu	Human adenovirus 5	96%	MF358604.1	Pos	Neg
30.	78 years	Sibu	Human adenovirus 5	98%	MF358604.1	Pos	Neg

^a^Specimens at Duke-NUS not run by conventional AdV-Hexon assay

**Table 4:** Human Adenovirus 7 Infections Detected at Sibu Hospital

Gender	Age (months)	Specimen Collection	Coinfection
Male	20	2/6/18	
Female	25	2/9/18	RSV-B
Male	9	3/7/18	
Male	25	3/22/18	
Female	9	4/27/18	RSV-B
Male	8	4/30/18	
Female	7	4/30/18	
Female	16	4/30/18	RSV-B
Male	6	5/4/18	EV
Female	3	5/4/18	
Male	4	5/4/18	
Male	20	5/4/18	RSV-B
Female	3	5/7/18	RSV-A
Male	7	3/7/18	

The NP specimens of two pediatric patients, both enrolled on May 3, 2018 at Sibu Hospital, were found to have 100% identity with the DrAdV1/PGT-0342 DNA polymerase gene, partial cds., *Desmondus rotundus* adenovirus 1, an AdV previously only detected in the common vampire bat. Both specimens were positive by all three assays at Duke University. Following sequencing, the two specimens were shipped to the University of Florida for attempts at viral isolation. AdV was not detected by PCR in spent cell culture media or cells inoculated with patient specimens.

We conducted next generation sequencing (Illumina iSeq100 and Nextera DNA Flex Library Prep Kit) on the two original clinical specimens as a potential new approach for clinical laboratory diagnostics. We submitted the resultant sequence data to Chan Zuckerberg Biohub’s IDseq metagenomics software pipeline [21]. However, our collaborators at IDseq found sparse DNA, not enough adenovirus-like reads to assemble a full genome. Hence, we were unable to determine with convincing rigor what might have given us the original novel conventional RT-PCR sequencing findings.

Of specimens found positive by the panspecies CoV assay at Duke University, only one was successfully sequenced. The sequenced specimen, which shared 93.7% identity with Human CoV OC43, was negative by rRT-PCR. The two positive specimens by the CoV panspecies at Duke-NUS were both sequenced with identity scores of 98.8 and 99.8% with Human CoV 229E.

Of the specimens successfully sequenced for HRV, HRV C was the most prevalent species (51%) followed by HRV A (41%) and HRV B (8%). Of the positive EV-panspecies specimens, 17 were successfully sequenced. Sequencing revealed three EV-71 specimens, all three collected from pediatric patients, two from Sibu Hospital and one from Kapit. There was also one EV-D68 detected and one coxsackievirus B5, also both collected from pediatric patients.

Agreement between the real-time and conventional PCR/RT-PCR assays were not as strong as we expected: adenovirus 87.2% agreement, Kappa 0.37; enterovirus 88.4% agreement, Kappa − 0.00; and coronavirus 91.8% agreement, Kappa 0.06 (see Supplemental Tables 6, 7, 8).

## Discussion

Based on positivity alone, the AdV, EV, and CoV panspecies assays found in total an additional 135 positive detections that were not found positive by real-time PCR. Only two specimens were positive by both the conventional and real-time CoV assays, with an additional 45 detections by the panspecies CoV assay, including a specimen that was successfully sequenced with 93.7% identity with CoV OC43 (see Supplementary Tables 5, 8). Similarly, only two of the specimens were positive by both the conventional and real-time EV assays, with 48 additional detections by the panspecies EV assay (see Supplementary Table 4,7). These findings would suggest that where possible, it is advisable to supplement real-time assay results with conventional molecular methods; however, we recognize the challenges to running conventional assays as compared to real-time.

The discrepancies between the AdV assays were greater with the AdV-Hex conventional assay than the rPCR assay. Comparing the results of the 30 successfully sequenced specimens positive by the panspecies assay, the rPCR assay detected 26 of the 30 positive specimens (84% agreement) (Table 3) while the conventional Adv-Hexon assay (only run on 26 of the 30 specimens) detected 15 of the positive specimens (53% agreement).

The 51 specimens positive by gel electrophoresis and sequence analysis for HRV were not the same as the 48 specimens positive by panspecies enterovirus assays (see Supplementary Table 4); however, we recognize the potential for cross-reactivity of the enterovirus primers as a potential limitation of the study. Additionally, during sequencing, 13 AdV specimens and two CoV specimens cross amplified human nucleic acid (see Supplementary Tables 3, 5).

As real-time PCR and RT-PCR have become gold standards in molecular diagnostics, conventional molecular methods are used less frequently due largely to their added complexity and time requirements. In a comparative study of real-time PCR versus other conventional detection methods such as cultures, urine antigen assays, and serologic tests, Yoshii et al. found that among adult community-acquired pneumonia patients, real-time PCR was significantly better able to detect both viral and bacterial pathogens in NP and sputum specimens compared to other conventional methods (72% pathogen detection rate versus 57%) [22]. However, had we relied exclusively on the rPCR and rRT-PCR results to select which specimens to sequence, we would have had an incomplete understanding of the epidemiology of these three viruses contributing to the burden of LRTIs in these two hospitals. The discordance detected in the results supports the decision to conduct both real-time and conventional PCR on these specimens, as these findings complemented one another.

This study had a number of limitations. Patient selection was dependent upon the willingness of clinicians to refer their patients for study, and the willingness of patients to enroll. Hence, the participants should be viewed as a convenience sample. Some of the differences in detection between molecular methods may be attributed to differences in the analytic sensitivities of the molecular methods. Our study was limited to a panel of viruses and therefore we were unable to determine the prevalence of bacterial mono- or coinfections within the population. We were not able to ascertain virus viability in the remote setting (Sarawak, Malaysia) as no virus culture capability was available. Because of multiple freeze thaw cycles, we only pursued live virus rescue for the two specimens with molecular evidence of a novel bat-like adenovirus. As described, those culture efforts failed. Hence, we were uncertain which of our molecular detections reflect live virus infections. A number of the samples detected positive by conventional assays were not successfully sequenced, potentially because the amount of starting material was suboptimal. Additionally, while our two laboratory teams (Singapore and USA) closely followed the same written procedures, there might have been differences in technique that contributed to inter-laboratory ascertainment biases. Even with these limitations it seems clear that the panspecies approaches for these three virus types were a more sensitive method.

## Conclusions

Through the panspecies conventional PCR/RT-PCR assays, we were able to detect LRTI that may have otherwise been missed with the rPCR/rRT-PCR assays. This was especially true for adenoviruses, with 44 specimens positive by the conventional HAdV panspecies assay that were negative by rPCR, which we would otherwise have been missed. Two specimens sharing 97% and 98% identity with human mastadenovirus C were negative by rPCR but positive by the panspecies assay and successfully sequenced. Another specimen, sharing 98% identity with human HAdV-41 was successfully sequenced after a positive panspecies assay result but was negative by rPCR. Hence, we conclude that where a sensitivity problem may be suspected, real-time assay results might be supplemented with conventional molecular methods.

## Supplementary information

**Additional file 1: Table S1.** Inclusion and exclusion criteria checklist used by medical officers while assessing patients for enrollment eligibility. **Table S2.** Demographic characteristics and exposure variables reported upon enrollment among patients hospitalized with pneumonia at Sibu and Kapit hospitals, between June 2017 and May 2018. **Table S3.** All specimens positive by one or more molecular method demonstrating discrepancies across adenovirus (AdV) assays. **Table S4.** All specimens positive by one or more molecular method demonstrating discrepancies across enterovirus (EV) assays. **Table S5.** All specimens positive by one or more molecular method demonstrating discrepancies across coronavirus (CoV) assays. **Table S6.** Comparison of positivity of panspecies conventional PCR versus real-time PCR for AdV. **Table S7.** Comparison of positivity of panspecies conventional PCR versus real-time PCR for EV. **Table S8.** Comparison of positivity of panspecies conventional PCR versus real-time PCR for CoV.

## Data Availability

The data collected during the current study are available from the corresponding author on reasonable request.

## Abbreviations

AdV: Adenovirus
BLAST: Basic Local Alignment Search Tool software
CoV: Coronavirus
EV: Enterovirus
HRV: Human rhinovirus
LRTI: Lower respiratory tract infections
NCBI: National Center for Biotechnology Information
NP: Nasopharyngeal
PCR: Polymerase chain reaction
RSV: Respiratory syncytial virus
rPCR: Real-time polymerase chain reaction
rRT-PCR: Real-time reverse-transcription polymerase chain reaction
SHCRC: Sibu Hospital Clinical Research Center
