# Supplementary material for: Panspecies molecular assays detect viral pathogens missed by real-time PCR/reverse-transcriptase PCR among pneumonia patients, Sarawak, Malaysia
Source: Trop Dis Travel Med Vaccines. 2020 Aug 12;6:13. doi: 10.1186/s40794-020-00114-2 (PMC7422451; doi:10.1186/s40794-020-00114-2)
Supplement: Supplementary file 1 — Additional file 1: Table S1. Inclusion and exclusion criteria checklist used by medical officers while assessing patients for enrollment eligibility. Table S2. Demographic characteristics and exposure variables reported upon enrollment among patients hospitalized with pneumonia at Sibu and Kapit hospitals, between June 2017 and May 2018. Table S3. All specimens positive by one or more molecular method demonstrating discrepancies across adenovirus (AdV) assays. Table S4. All specimens positive by one or more molecular method demonstrating discrepancies across enterovirus (EV) assays. Table S5. All specimens positive by one or more molecular method demonstrating discrepancies across coronavirus (CoV) assays. Table S6. Comparison of positivity of panspecies conventional PCR versus real-time PCR for AdV. Table S7. Comparison of positivity of panspecies conventional PCR versus real-time PCR for EV. Table S8. Comparison of positivity of panspecies conventional PCR versus real-time PCR for CoV. [file 40794_2020_114_MOESM1_ESM.docx]

**Supplemental Appendix**

**Panspecies Molecular Assays Detect Viral Pathogens Missed by Real-Time PCR/Reverse-Transcriptase PCR Among Pneumonia Patients, Sarawak, Malaysia**

Jane K. Fieldhouse^1,2,3^, Emily S. Bailey^1,2,4^, Teck-Hock Toh^5,6,7^, King-Ching Hii^8^, Kerry A. Mallinson^2^, Jakie Ting^5,6^, John A. Lednicky^9,10^, Antoinette Berita^8^, Tham Thi Nguyen^11^, Diego Galan Donlo^1,11^, Son T. Than^11^, See-Chang Wong^6,7^, Toh-Mee Wong^5,6,12^, Patrick J. Blair^13^,

and Gregory C. Gray^1,2,11,14^

^1^Division of Infectious Diseases, Duke University School of Medicine, Durham, North Carolina, USA

^2^Duke Global Health Institute, Duke University, Durham, North Carolina, USA

^3^Institute for Global Health Sciences, University of California, San Francisco, California, USA

^4^Department of Public Health, Texas Tech University Health Sciences Center, Abilene, Texas, USA

^5^Clinical Research Center, Sibu Hospital, Ministry of Health Malaysia, Sibu, Sarawak, Malaysia

^6^Faculty of Medicine, SEGi University, Kota Damansara, Selangor, Malaysia

^7^Department of Paediatrics, Sibu Hospital, Ministry of Health Malaysia, Sibu, Sarawak, Malaysia

^8^Kapit Hospital, Ministry of Health Malaysia, Kapit, Sarawak, Malaysia

^9^Department of Environmental and Global Health, University of Florida, Gainesville, Florida, USA

^10^Emerging Pathogens Institute, University of Florida, Gainesville, Florida, USA

^11^Emerging Infectious Disease Program, Duke-NUS Medical School, Singapore

^12^Department of Medicine, Sibu Hospital, Ministry of Health Malaysia, Sibu, Sarawak, Malaysia

^13^Naval Medical Research Center-Asia, Singapore

^14^Global Health Center, Duke Kunshan University, Kunshan, China

Supplementary Table 1. Inclusion and exclusion criteria checklist used by medical officers while assessing patients for enrollment eligibility

| Inclusion Criteria Children (1 month to 18 years) | Inclusion Criteria Adults (18 years or more) |
| --- | --- |
| - They were admitted to Sibu or Kapit hospital; - Have evidence of acute infection, defined as reported fever or chills, documented fever or hypothermia, or leukocytosis or leukopenia; - Have evidence of an acute respiratory illness, defined as new cough or sputum production, chest pain, dyspnea, tachypnea, abnormal lung examination, or respiratory failure; - A parent or legal guardian provides written informed consent. In addition to parental consent, signed assent document will be sought from children 7 to 18 years of age. - The evidence of illness is consistent with pneumonia as assessed by means of chest radiography within 72 hours before or after admission. | - They were admitted to Sibu or Kapit hospital on the basis of a clinical assessment by the treating clinician; - Have evidence of acute infection, defined as reported fever or chills, documented fever or hypothermia, leukocytosis or leukopenia, or new altered mental status; - Have evidence of an acute respiratory illness, defined as new cough or sputum production, chest pain, dyspnea, tachypnea, abnormal lung examination, or respiratory failure; - Have evidence consistent with pneumonia as assessed by means of chest radiography by the clinical team within 48 hours before or after admission. |
| Exclusion Criteria Children (1 month to 18 years) | Exclusion Criteria Adults (18 years or more) |
| - If they had been hospitalized recently (<7 days for immunocompetent children and <90 days for immunosuppressed children) - If they had already been enrolled in this study within the previous 28 days - If they resided in an extended-care facility - If they had an alternative diagnosis of a respiratory disorder - If they are newborns who never left the hospital - If they have a tracheostomy tube - If they have cystic fibrosis or - If they have cancer with neutropenia - If they have received a solid-organ or hematopoietic stem-cell transplant within the previous 90 days - If they have active graft-versus-host disease or bronchiolitis obliterans - If they have human immunodeficiency virus infection with a CD4 cell count of less than 200 per cubic millimeter (or a percentage of CD4 cells <14%). | - If they had been hospitalized recently (<28 days for immunocompetent patients and <90 days for immunosuppressed patients), - If they have already been enrolled in this study within the previous 28 days - If they were functionally dependent nursing home residents, - If they have a clear alternative diagnosis - If they have undergone tracheotomy - If they have a percutaneous endoscopic gastrostomy tube - If they have cystic fibrosis - If they have cancer with neutropenia, - If they have received a solid-organ or hematopoietic stem-cell transplant within the previous 90 days, - If they have active graft-versus-host disease - If they have bronchiolitis obliterans - If they have human immunodeficiency virus infection with a CD4 cell count of less than 200 per cubic millimeter. |

Adapted from: Jain S et al. 2015;372(9) [1] and Jain S et al. 2015;373(5) [2].

Supplementary Table 2. Demographic characteristics and exposure variables reported upon enrollment among patients hospitalized with pneumonia at Sibu and Kapit hospitals, between June 2017 and May 2018

| Exposure variables | All Total No. (%) | Sibu No. (%) | Kapit No. (%) |  |
| --- | --- | --- | --- | --- |
| Total | 600 (100) | 389 (64.8) | 211 (35.2) |  |
| Gender |  |  |  |  |
| Female | 274 (45.7) | 187 (48.1) | 87 (41.2) |  |
| Male | 326 (54.3) | 202 (51.9) | 124 (58.8) |  |
| Age group (Approx.^*^ Quartiles) |  |  |  |  |
| 0-1 years (µ 0.5) | 180 (30.0) | 93 (23.9) | 87 (41.2) |  |
| 1-2 years (µ 1.4) | 105 (17.5) | 60 (15.4) | 45 (21.3) |  |
| 2-18 years (µ 6.4) | 154 (25.7) | 94 (24.2) | 60 (28.4) |  |
| >18 years (µ 63.9) | 161 (26.8) | 142 (36.5) | 19 (9.0) |  |
| Household size (Quartiles) |  |  |  |  |
| 0-3 (µ 2.0) | 161 (26.8) | 113 (29.0) | 48 (22.8) |  |
| 4-5 (µ 4.5) | 178 (29.7) | 126 (32.4) | 52 (24.6) |  |
| 6-7 (µ 6.4) | 133 (22.2) | 71 (18.3) | 62 (29.4) |  |
| ≥ 8 (µ 11.2) | 128 (21.3) | 79 (20.3) | 49 (23.2) |  |
| Ethnicity |  |  |  |  |
| Iban | 441 (73.5) | 253 (65.0) | 188 (89.1) |  |
| Malay | 42 (7.0) | 36 (9.3) | 6 (2.8) |  |
| Chinese | 46 (7.7) | 43 (11.1) | 3 (1.4) |  |
| Other | 71 (11.8) | 57 (14.7) | 14 (06.6) |  |
| Pre-existing conditions |  |  |  |  |
| Hypertension | 89 (14.8) | 76 (19.5) | 13 (6.2) |  |
| Diabetes mellitus | 37 (6.2) | 31 (8.0) | 6 (2.8) |  |
| Lung disease | 116 (19.3) | 112 (28.8) | 4 (1.9) |  |
| Other | 60 (10.0) | 54 (13.9) | 6 (2.8) |  |
| None | 389 (64.8) | 197 (50.6) | 192 (91.0) |  |
| Medical treatment in last 6 months |  |  |  |  |
| Diabetes medicine | 36 (6.0) | 30 (6.5) | 6 (3.4) |  |
| Hypertension medicine | 84 (14.0) | 73 (14.3) | 11 (12.5) |  |
| Corticosteroids | 93 (15.5) | 86 (22.1) | 7 (3.3) |  |
| Other | 48 (8.0) | 47 (12.1) | 1 (0.5) |  |
| None | 405 (67.5) | 211 (54.2) | 194 (91.9) |  |
| Animal contact^†^ |  |  |  |  |
| Pigs | 18 (3.0) | 15 (3.9) | 3 (1.4) |  |
| Chickens | 68 (11.3) | 41 (10.5) | 27 (12.8) |  |
| Ducks and other poultry | 33 (5.5) | 11 (2.8) | 2 (1.0) |  |
| Cats | 141 (23.7) | 117 (30.0) | 24 (11.4) |  |
| Dogs | 73 (12.2) | 57 (14.7) | 16 (7.6) |  |
| Other^‡^ | 177 (29.5) | 144 (37.0) | 33 (15.6) |  |
| None | 326 (54.3) | 117 (30.1) | 149 (70.6) |  |
| Abbreviations: COPD, Chronic Obstructive Pulmonary Disease; µ, mean age within category  ^*^Rounded to whole years based on age quartiles: 0.06-0.85 (Q1); 0.86-2.16 (Q2); 2.17-25.2 (Q3); 25.3-92 (Q4)  ^†^Animal contact defined as touched or come within one meter in the last 30 days | | | | |

**^‡^**Other animals include: Cow, rats, rabbits, a snake, and a monkey

| Supplementary Table 3. All specimens positive by one or more molecular method demonstrating discrepancies across adenovirus assays | | | | | | | |
| --- | --- | --- | --- | --- | --- | --- | --- |
| Specimen ID | Hospital | qPCR AdV Assay | Panspecies Conventional Adv Assay | Identity Score | Accession Number | AdV-Hexon Assay | Comment |
| 06 | Sibu | Neg | Neg | -- | -- | Pos |  |
| 15 | Sibu | Pos | Neg | -- | -- | Pos |  |
| 49 | Sibu | Pos | Neg | -- | -- | Neg |  |
| 52 | Sibu | Neg | Pos | -- | -- | Neg | Sequencing not successful |
| 59 | Sibu | Neg | Pos | -- | -- | Neg | Sequencing not successful |
| 64 | Sibu | Neg | Pos | -- | -- | Neg | No similarity |
| 66 | Sibu | Neg | Pos | -- | -- | Neg | Human DNA |
| 68 | Sibu | Neg | Pos | -- | -- | Neg | Human DNA |
| 70 | Sibu | Neg | Pos | -- | -- | Neg | Human DNA |
| 71 | Sibu | Neg | Pos | -- | -- | Neg | Human DNA |
| 72 | Sibu | Neg | Pos | -- | -- | Neg | Human DNA |
| 73 | Sibu | Neg | Pos | -- | -- | Neg | Human DNA |
| 75 | Sibu | Neg | Pos | -- | -- | Neg | Human DNA |
| 76 | Sibu | Neg | Human mastadenovirus C | 97% | MH121097.1 | Neg |  |
| 82 | Sibu | Pos | Human adenovirus C | 95% | MF358574.1 | Not performed | Specimens at Duke-NUS |
| 100 | Kapit | Neg | Human mastadenovirus C | 98% | MH121117.1 | Neg |  |
| 112 | Kapit | Pos | Human mastadenovirus E | 99% | KY996453.1 | Pos |  |
| 116 | Kapit | Neg | Neg | -- | -- | Pos |  |
| 128 | Kapit | Pos | Neg | -- | -- | Neg |  |
| 134 | Kapit | Pos | Pos | -- | -- | Neg | Sequencing not successful |
| 138 | Kapit | Pos | Human mastadenovirus C | 98% | MH121117.1 | Pos |  |
| 141 | Kapit | Neg | Neg | -- | -- | Pos |  |
| 145 | Kapit | Pos | Neg | -- | -- | Neg |  |
| 146 | Kapit | Neg | Neg | -- | -- | Pos |  |
| 1032 | Sibu | Pos | Human adenovirus C | 98% | MF358562.1 | Not performed | Specimens at Duke-NUS |
| 1040 | Sibu | Pos | Neg | -- | -- | Not performed | Specimens at Duke-NUS |
| 1056 | Sibu | Neg | Neg | -- | -- | Pos |  |
| 1074 | Sibu | Neg | Neg | -- | -- | Pos |  |
| 1082 | Sibu | Pos | Neg | -- | -- | Pos |  |
| 1083 | Sibu | Pos | Neg | -- | -- | Pos |  |
| 1084 | Sibu | Pos | Neg | -- | -- | Pos |  |
| 1085 | Sibu | Neg | Neg | -- | -- | Pos |  |
| 1086 | Sibu | Pos | Neg | -- | -- | Pos |  |
| 1087 | Sibu | Suspect | Neg | -- | -- | Pos |  |
| 1088 | Sibu | Neg | Neg | -- | -- | Pos |  |
| 1090 | Sibu | Pos | Neg | -- | -- | Neg |  |
| 1091 | Sibu | Pos | Human adenovirus B | 99% | KF268212.1 | Neg |  |
| 1092 | Sibu | Pos | Human adenovirus 7 | 99% | MG923582.1 | Neg |  |
| 1093 | Sibu | Pos | Neg | -- | -- | Pos |  |
| 1097 | Sibu | Pos | Human adenovirus 7 | 98% | MG923582.1 | Neg |  |
| 1100 | Sibu | Neg | Pos | -- | -- | Neg | Sequencing not successful |
| 1101 | Sibu | Neg | Pos | -- | -- | Neg | Sequencing not successful |
| 1106 | Sibu | Neg | Pos | -- | -- | Neg | Sequencing not successful |
| 1107 | Sibu | Neg | Pos | -- | -- | Neg | Sequencing not successful |
| 1109 | Sibu | Neg | Pos | -- | -- | Neg | Sequencing not successful |
| 1114 | Sibu | Neg | Pos | -- | -- | Neg | Sequencing not successful |
| 1115 | Sibu | Pos | Neg | -- | -- | Neg |  |
| 1116 | Sibu | Pos | Human adenovirus 7 | 98% | MG923582.1 | Neg |  |
| 1117 | Sibu | Pos | Neg | -- | -- | Neg |  |
| 1121 | Sibu | Neg | Neg | -- | -- | Pos |  |
| 1124 | Sibu | Pos | Human adenovirus 7 | 99% | MG923582.1 | Pos |  |
| 1125 | Sibu | Neg | Pos | -- | -- | Pos | Sequencing not successful |
| 1127 | Sibu | Neg | Neg | -- | -- | Pos |  |
| 1128 | Sibu | Neg | Neg | -- | -- | Pos |  |
| 1130 | Sibu | Neg | Neg | -- | -- | Pos |  |
| 1131 | Sibu | Neg | Neg | -- | -- | Pos |  |
| 1133 | Sibu | Pos | Neg | -- | -- | Pos |  |
| 1135 | Sibu | Neg | Neg | -- | -- | Pos |  |
| 1136 | Sibu | Neg | Neg | -- | -- | Pos |  |
| 1137 | Sibu | Pos | Human adenovirus 5 | 99% | MF358604.1 | Neg |  |
| 1138 | Sibu | Neg | Neg | -- | -- | Pos |  |
| 1139 | Sibu | Pos | Neg | -- | -- | Pos |  |
| 1140 | Sibu | Pos | Neg | -- | -- | Pos |  |
| 1141 | Sibu | Neg | Neg | -- | -- | Pos |  |
| 1142 | Sibu | Neg | Neg | -- | -- | Pos |  |
| 1143 | Sibu | Pos | Neg | -- | -- | Pos |  |
| 1145 | Sibu | Pos | Neg | -- | -- | Pos |  |
| 1146 | Sibu | Neg | Neg | -- | -- | Pos |  |
| 1147 | Sibu | Neg | Neg | -- | -- | Pos |  |
| 1148 | Sibu | Neg | Neg | -- | -- | Pos |  |
| 1149 | Sibu | Neg | Neg | -- | -- | Pos |  |
| 1150 | Sibu | Neg | Neg | -- | -- | Pos |  |
| 1151 | Sibu | Neg | Neg | -- | -- | Pos |  |
| 1152 | Sibu | Neg | Neg | -- | -- | Pos |  |
| 1153 | Sibu | Neg | Neg | -- | -- | Pos |  |
| 1154 | Sibu | Pos | Neg | -- | -- | Pos |  |
| 1156 | Sibu | Neg | Neg | -- | -- | Pos |  |
| 1157 | Sibu | Pos | Neg | -- | -- | Pos |  |
| 1158 | Sibu | Pos | Human Adenovirus 7 | 98% | MG923582.1 | Pos |  |
| 1159 | Sibu | Pos | Human Adenovirus 7 | 98% | MG923582.1 | Pos |  |
| 1160 | Sibu | Pos | Human Adenovirus 7 | 98% | MG923582.1 | Pos |  |
| 1161 | Sibu | Pos | Human Adenovirus 7 | 97% | MG923582.1 | Pos |  |
| 1162 | Sibu | Pos | Desmondus rotundus Adenovirus 1 | 100% | KX774303.1 | Pos |  |
| 1163 | Sibu | Pos | Desmondus rotundus Adenovirus 1 | 100% | KX774303.1 | Pos |  |
| 1164 | Sibu | Pos | Human Adenovirus 7 | 96% | MG923582.1 | Pos |  |
| 1165 | Sibu | Pos | Human Adenovirus 7 | 93% | MG923582.1 | Pos |  |
| 1166 | Sibu | Pos | Human DNA |  |  | Pos |  |
| 1167 | Sibu | Pos | Human Adenovirus 7 | 98% | MG923582.1 | Pos |  |
| 1168 | Sibu | Pos | Human DNA | 97% | AL591128.7 | Pos |  |
| 1169 | Sibu | Pos | Human Adenovirus 7 | 98% | MG923582.1 | Pos |  |
| 1170 | Sibu | Pos | Neg | -- | -- | Pos |  |
| 1171 | Sibu | Pos | Human Adenovirus 7 | 97% | MG923582.1 | Pos |  |
| 1172 | Sibu | Pos | Neg | -- | -- | Pos |  |
| 1175 | Sibu | Neg | Neg | -- | -- | Pos |  |
| 1176 | Sibu | Pos | Neg | -- | -- | Neg |  |
| 1177 | Sibu | Neg | Neg | -- | -- | Pos |  |
| 1178 | Sibu | Neg | Neg | -- | -- | Pos |  |
| 1182 | Sibu | Pos | Pos | -- | -- | Neg | Sequencing not successful |
| 2062 | Sibu | Neg | Neg | -- | -- | Pos |  |
| 2068 | Sibu | Neg | Neg | -- | -- | Pos |  |
| 2081 | Sibu | Neg | Neg | -- | -- | Pos |  |
| 2084 | Sibu | Pos | Human adenovirus 4 | 99% | AP014851.1 | Pos |  |
| 2103 | Sibu | Pos | Neg | -- | -- | Neg |  |
| 2115 | Sibu | Pos | Human adenovirus 5 | 96% | MF358604.1 | Neg |  |
| 2119 | Sibu | Pos | Human adenovirus 5 | 98% | MF358604.1 | Neg |  |
| 3012 | Kapit | Pos | Neg | -- | -- | Not performed | Specimens at Duke-NUS |
| 3013 | Kapit | Neg | Pos | -- | -- | Not performed | Sequencing not successful; specimens at Duke-NUS |
| 3016 | Kapit | Pos | Neg | -- | -- | Not performed | Specimens at Duke-NUS |
| 3018 | Kapit | Pos | Neg | -- | -- | Not performed | Specimens at Duke-NUS |
| 3025 | Kapit | Neg | Human adenovirus 41 | 98% | KX868523.2 | Not performed | Specimens at Duke-NUS |
| 3037 | Kapit | Pos | Neg | -- | -- | Not performed | Specimens at Duke-NUS |
| 3042 | Kapit | Neg | Pos | -- | -- | Not performed | Sequencing not successful; specimens at Duke-NUS |
| 3043 | Kapit | Neg | Pos | -- | -- | Not performed | Sequencing not successful; specimens at Duke-NUS |
| 3044 | Kapit | Neg | Pos | -- | -- | Not performed | Sequencing not successful; specimens at Duke-NUS |
| 3046 | Kapit | Neg | Pos | -- | -- | Not performed | Sequencing not successful; specimens at Duke-NUS |
| 3048 | Kapit | Neg | Pos | -- | -- | Not performed | Sequencing not successful; specimens at Duke-NUS |
| 3050 | Kapit | Neg | Pos | -- | -- | Not performed | Sequencing not successful; specimens at Duke-NUS |
| 3051 | Kapit | Neg | Pos | -- | -- | Not performed | Sequencing not successful; specimens at Duke-NUS |
| 3054 | Kapit | Neg | Pos | -- | -- | Not performed | Sequencing not successful; specimens at Duke-NUS |
| 3055 | Kapit | Neg | Pos | -- | -- | Not performed | Sequencing not successful; specimens at Duke-NUS |
| 3056 | Kapit | Neg | Pos | -- | -- | Not performed | Sequencing not successful; specimens at Duke-NUS |
| 3058 | Kapit | Neg | Pos | -- | -- | Not performed | Sequencing not successful; specimens at Duke-NUS |
| 3063 | Kapit | Neg | Neg | -- | -- | Pos |  |
| 3071 | Kapit | Pos | Neg | -- | -- | Pos |  |
| 3077 | Kapit | Neg | Neg | -- | -- | Pos |  |
| 3079 | Kapit | Pos | Human adenovirus 5 | 98% | MF358601.1 | Pos |  |
| 3082 | Kapit | Neg | Neg | -- | -- | Pos |  |
| 3090 | Kapit | Neg | Neg | -- | -- | Pos |  |
| 3094 | Kapit | Neg | Neg | -- | -- | Pos |  |
| 3119 | Kapit | Pos | Neg | -- | -- | Neg |  |
| 3124 | Kapit | Neg | Neg | -- | -- | Pos |  |
| 3125 | Kapit | Neg | Neg | -- | -- | Pos |  |
| 3126 | Kapit | Pos | Human adenovirus 7 | 97% | MG923582.1 | Pos |  |
| 3128 | Kapit | Neg | Neg | -- | -- | Pos |  |
| 3130 | Kapit | Neg | Neg | -- | -- | Pos |  |
| 3131 | Kapit | Neg | Neg | -- | -- | Pos |  |
| 3135 | Kapit | Pos | Neg | -- | -- | Neg |  |
| 3136 | Kapit | Neg | Neg | -- | -- | Pos |  |
| 3138 | Kapit | Neg | Neg | -- | -- | Pos |  |
| 3139 | Kapit | Neg | Pos | -- | -- | Neg | Human DNA |
| 3141 | Kapit | Neg | Pos | -- | -- | Neg | Human DNA |
| 3143 | Kapit | Neg | Pos | -- | -- | Neg | Sequencing not successful |
| 3144 | Kapit | Neg | Pos | -- | -- | Neg | Sequencing not successful |
| 3145 | Kapit | Neg | Pos | -- | -- | Neg | Human DNA |
| 3147 | Kapit | Neg | Pos | -- | -- | Neg | Human DNA |
| 3148 | Kapit | Neg | Pos | -- | -- | Neg | Human DNA |
| 3149 | Kapit | Neg | Pos | -- | -- | Pos | Sequencing not successful |
| 3150 | Kapit | Pos | Neg | -- | -- | Pos |  |
| 3153 | Kapit | Neg | Pos | -- | -- | Neg | Sequencing not successful |
| 3154 | Kapit | Neg | Pos | -- | -- | Neg | Human DNA |
| 3155 | Kapit | Neg | Pos | -- | -- | Neg | Sequencing not successful |
| 3157 | Kapit | Neg | Pos | -- | -- | Neg | Sequencing not successful |

Supplementary Table 4. All specimens positive by one or more molecular method demonstrating discrepancies across enterovirus

(EV) assays

| Specimen ID | Hospital | qPCR EV Assay | Panspecies Conventional EV Assay | Identity Score | Accession Number | Rhinovirus Assay | Comment |
| --- | --- | --- | --- | --- | --- | --- | --- |
| 07 | Sibu | Neg | Neg | -- | -- | HRV C |  |
| 11 | Sibu | Neg | Neg | -- | -- | HRV A |  |
| 14 | Sibu | Neg | Pos | -- | -- | Neg | Sequencing not successful |
| 20 | Sibu | Pos | Neg | -- | -- | Neg |  |
| 36 | Sibu | Neg | Neg | -- | -- | HRV A |  |
| 39 | Sibu | Neg | Human enterovirus 71 | 94% | JN874547.1 | Neg |  |
| 40 | Sibu | Neg | Pos | -- | -- | Neg | Sequencing not successful |
| 41 | Sibu | Pos | Neg | -- | -- | Neg |  |
| 52 | Sibu | Pos | Neg | -- | -- | HRV C |  |
| 72 | Sibu | Neg | Pos | -- | -- | Neg | Sequencing not successful |
| 107 | Kapit | Pos | Neg | -- | -- | Neg |  |
| 123 | Kapit | Pos | Neg | -- | -- | Neg |  |
| 135 | Kapit | Pos | Neg | -- | -- | Neg |  |
| 138 | Kapit | Pos | Neg | -- | -- | Neg |  |
| 141 | Kapit | Pos | Neg | -- | -- | Neg |  |
| 145 | Kapit | Pos | Neg | -- | -- | Neg |  |
| 1010 | Sibu | Pos | Neg | -- | -- | Not performed |  |
| 1012 | Sibu | Pos | Coxsackievirus B5 | 97% | MF589295.1 | Not performed |  |
| 1023 | Sibu | Neg | Pos | -- | -- | Not performed | Sequencing not successful |
| 1024 | Sibu | Neg | Pos | -- | -- | Not performed | Sequencing not successful |
| 1025 | Sibu | Neg | Pos | -- | -- | Not performed | Sequencing not successful |
| 1026 | Sibu | Neg | Pos | -- | -- | Not performed | Sequencing not successful |
| 1032 | Sibu | Neg | Rhinovirus B | 93% | KP736642.1 | Not performed |  |
| 1033 | Sibu | Neg | Pos | -- | -- | Not performed | Sequencing not successful |
| 1049 | Sibu | Neg | Enterovirus 71 | 81% | HQ283818.1 | Not performed |  |
| 1051 | Sibu | Neg | Rhinovirus 14 | 89% | AY355195.1 | Not performed |  |
| 1054 | Sibu | Neg | Neg | -- | -- | HRV C |  |
| 1056 | Sibu | Pos | Neg | -- | -- | Neg |  |
| 1057 | Sibu | Neg | Neg | -- | -- | HRV A |  |
| 1063 | Sibu | Neg | Neg | -- | -- | HRV C |  |
| 1068 | Sibu | Pos | Neg | -- | -- | HRV C |  |
| 1070 | Sibu | Neg | Neg | -- | -- | HRV C |  |
| 1073 | Sibu | Pos | Neg | -- | -- | HRV C |  |
| 1074 | Sibu | Neg | Neg | -- | -- | HRV A |  |
| 1075 | Sibu | Pos | Neg | -- | -- | Neg |  |
| 1080 | Sibu | Neg | Pos | -- | -- | Neg | Sequencing not successful |
| 1085 | Sibu | Neg | Pos | -- | -- | Neg | Sequencing not successful |
| 1090 | Sibu | Neg | Pos | -- | -- | Neg | Sequencing not successful |
| 1096 | Sibu | Neg | Pos | -- | -- | Neg | Sequencing not successful |
| 1098 | Sibu | Neg | Pos | -- | -- | Neg | Sequencing not successful |
| 1100 | Sibu | Neg | Neg | -- | -- | HRV A |  |
| 1101 | Sibu | Neg | Neg | -- | -- | HRV A |  |
| 1102 | Sibu | Neg | Neg | -- | -- | HRV A |  |
| 1104 | Sibu | Neg | Pos | -- | -- | Neg | Sequencing not successful |
| 1108 | Sibu | Neg | Pos | -- | -- | Neg | Sequencing not successful |
| 1111 | Sibu | Neg | Pos | -- | -- | Neg | Sequencing not successful |
| 1113 | Sibu | Neg | Pos | -- | -- | Neg | Sequencing not successful |
| 1118 | Sibu | Neg | Pos | -- | -- | Neg | Sequencing not successful |
| 1123 | Sibu | Neg | Pos | -- | -- | Neg | Sequencing not successful |
| 1124 | Sibu | Neg | Human rhinovirus A22 | 79% | KY342346.1 | Neg |  |
| 1126 | Sibu | Neg | Pos | -- | -- | Neg | Sequencing not successful |
| 1131 | Sibu | Neg | Pos | -- | -- | Neg | Sequencing not successful |
| 1134 | Sibu | Neg | Pos | -- | -- | Neg | Sequencing not successful |
| 1137 | Sibu | Neg | Neg | -- | -- | HRV A |  |
| 1143 | Sibu | Neg | Pos | -- | -- | Neg | Sequencing not successful |
| 1147 | Sibu | Neg | Neg | -- | -- | HRV C |  |
| 1151 | Sibu | Neg | Pos | -- | -- | Neg | Sequencing not successful |
| 1153 | Sibu | Neg | Neg | -- | -- | HRV C |  |
| 1154 | Sibu | Pos | Neg | -- | -- | Neg |  |
| 1164 | Sibu | Pos | Neg | -- | -- | Neg |  |
| 1170 | Sibu | Neg | Neg | -- | -- | HRV B 70 |  |
| 2031 | Sibu | Neg | Rhinovirus A | 99% | KP737142.1 | Not performed |  |
| 2033 | Sibu | Neg | Pos | -- | -- | Not performed | Sequencing not successful |
| 2034 | Sibu | Neg | Pos | -- | -- | Not performed | Sequencing not successful |
| 2037 | Sibu | Neg | Pos | -- | -- | Not performed | Sequencing not successful |
| 2039 | Sibu | Neg | Pos | -- | -- | Not performed | Sequencing not successful |
| 2056 | Sibu | Neg | Neg | -- | -- | HRV C |  |
| 2064 | Sibu | Neg | Neg | -- | -- | HRV C |  |
| 2070 | Sibu | Pos | Neg | -- | -- | HRV C |  |
| 2092 | Sibu | Pos | Neg | -- | -- | HRV A |  |
| 2112 | Sibu | Pos | Neg | -- | -- | HRV A |  |
| 2114 | Sibu | Pos | Neg | -- | -- | HRV A |  |
| 3005 | Kapit | Neg | Pos | -- | -- | Not performed | Sequencing not successful |
| 3006 | Kapit | Neg | Enterovirus 71 | 97% | JN544418.1 | Not performed |  |
| 3008 | Kapit | Neg | Rhinovirus A | 98% | KP737215.1 | Not performed |  |
| 3009 | Kapit | Neg | Rhinovirus A77 | 97% | MF161064.1 | Not performed |  |
| 3012 | Kapit | Pos | Neg | -- | -- | Not performed |  |
| 3013 | Kapit | Neg | Rhinovirus A | 96% | KP737146.1 | Not performed |  |
| 3017 | Kapit | Pos | Enterovirus D68 | 97% | KX789230.1 | Not performed |  |
| 3018 | Kapit | Pos | Neg | -- | -- | Not performed |  |
| 3021 | Kapit | Neg | Rhinovirus A | 96% | KP737146.1 | Not performed |  |
| 3032 | Kapit | Neg | Rhinovirus A | 94% | P737278.1 | Not performed |  |
| 3035 | Kapit | Neg | Rhinovirus A | 95% | MH685693.1 | Not performed |  |
| 3036 | Kapit | Neg | Rhinovirus A12 | 92% | KX398052.1 | Not performed |  |
| 3065 | Kapit | Neg | Neg | -- | -- | HRV A |  |
| 3076 | Kapit | Neg | Neg | -- | -- | HRV C |  |
| 3077 | Kapit | Neg | Neg | -- | -- | HRV A |  |
| 3079 | Kapit | Pos | Neg | -- | -- | HRV C |  |
| 3082 | Kapit | Neg | Neg | -- | -- | HRV A |  |
| 3085 | Kapit | Neg | Neg | -- | -- | HRV A |  |
| 3086 | Kapit | Pos | Neg | -- | -- | HRV B |  |
| 3093 | Kapit | Neg | Neg | -- | -- | HRV C |  |
| 3097 | Kapit | Neg | Neg | -- | -- | HRV C |  |
| 3100 | Kapit | Neg | Neg | -- | -- | HRV A |  |
| 3110 | Kapit | Pos | Neg | -- | -- | HRV C |  |
| 3112 | Kapit | Neg | Neg | -- | -- | HRV B |  |
| 3113 | Kapit | Neg | Pos | -- | -- | Neg | Sequencing not successful |
| 3122 | Kapit | Neg | Neg | -- | -- | HRV A |  |
| 3127 | Kapit | Neg | Neg | -- | -- | HRV A |  |
| 3128 | Kapit | Neg | Neg | -- | -- | HRV C |  |
| 3129 | Kapit | Neg | Neg | -- | -- | HRV C |  |
| 3132 | Kapit | Neg | Neg | -- | -- | HRV C |  |
| 3138 | Kapit | Pos | Neg | -- | -- | HRV A |  |
| 3139 | Kapit | Neg | Neg | -- | -- | HRV B |  |
| 3142 | Kapit | Pos | Neg | -- | -- | HRV C |  |
| 3147 | Kapit | Neg | Neg | -- | -- | HRV A |  |
| 3154 | Kapit | Neg | Neg | -- | -- | HRV A |  |
| 3155 | Kapit | Neg | Human rhinovirus A22 | 97% | KY342346.1 | Neg |  |
| 3159 | Kapit | Neg | Neg | -- | -- | HRV C |  |
| 3160 | Kapit | Neg | Neg | -- | -- | HRV C |  |
| 3161 | Kapit | Neg | Neg | -- | -- | HRV C |  |
| 3162 | Kapit | Neg | Neg | -- | -- | HRV C |  |

Supplementary Table 5. All specimens positive by one or more molecular method demonstrating discrepancies across coronavirus (CoV) assays

| Specimen ID | Hospital | qRT-PCR CoV Assay | Panspecies Conventional CoV Assay | Identity Score | Accession Number | Comment |
| --- | --- | --- | --- | --- | --- | --- |
| 08 | Sibu | Pos | Neg | -- | -- | Negative on rerun; sequencing not successful |
| 09 | Sibu | Neg | Human coronavirus OC43 | 94% | MN026164.1 | Negative on rerun |
| 34 | Sibu | Neg | Pos | -- | -- | Human DNA |
| 39 | Sibu | Neg | Pos | -- | -- | Sequencing not successful |
| 41 | Sibu | Neg | Pos | -- | -- | Sequencing not successful |
| 44 | Sibu | Neg | Pos | -- | -- | Sequencing not successful |
| 49 | Sibu | Neg | Pos | -- | -- | Sequencing not successful |
| 50 | Sibu | Neg | Pos | -- | -- | Sequencing not successful |
| 56 | Sibu | Neg | Pos | -- | -- | Sequencing not successful |
| 57 | Sibu | Neg | Pos | -- | -- | Sequencing not successful |
| 107 | Kapit | Neg | Pos | -- | -- | Sequencing not successful |
| 108 | Kapit | Neg | Pos | -- | -- | Sequencing not successful |
| 111 | Kapit | Neg | Pos | -- | -- | Sequencing not successful |
| 112 | Kapit | Neg | Pos | -- | -- | Sequencing not successful |
| 113 | Kapit | Neg | Pos | -- | -- | Sequencing not successful |
| 121 | Kapit | Neg | Pos | -- | -- | Sequencing not successful |
| 134 | Kapit | Neg | Pos | -- | -- | Sequencing not successful |
| 137 | Kapit | Neg | Pos | -- | -- | Sequencing not successful |
| 139 | Kapit | Neg | Pos | -- | -- | Sequencing not successful |
| 142 | Kapit | Neg | Pos | -- | -- | Sequencing not successful |
| 1110 | Sibu | Neg | Pos | -- | -- | Sequencing not successful |
| 1113 | Sibu | Neg | Pos | -- | -- | Sequencing not successful |
| 1115 | Sibu | Neg | Pos | -- | -- | Sequencing not successful |
| 1120 | Sibu | Neg | Pos | -- | -- | Sequencing not successful |
| 1122 | Sibu | Neg | Pos | -- | -- | Sequencing not successful |
| 1126 | Sibu | Neg | Pos | -- | -- | Sequencing not successful |
| 1127 | Sibu | Neg | Pos | -- | -- | Human DNA |
| 1128 | Sibu | Neg | Pos | -- | -- | Sequencing not successful |
| 1129 | Sibu | Neg | Pos | -- | -- | Sequencing not successful |
| 1133 | Sibu | Neg | Pos | -- | -- | Sequencing not successful |
| 1139 | Sibu | Neg | Pos | -- | -- | Sequencing not successful |
| 1140 | Sibu | Neg | Pos | -- | -- | Sequencing not successful |
| 1142 | Sibu | Neg | Pos | -- | -- | Sequencing not successful |
| 1146 | Sibu | Neg | Pos | -- | -- | Sequencing not successful |
| 1150 | Sibu | Neg | Pos | -- | -- | Sequencing not successful |
| 1151 | Sibu | Neg | Pos | -- | -- | Sequencing not successful |
| 1160 | Sibu | Neg | Pos | -- | -- | Sequencing not successful |
| 1178 | Sibu | Neg | Pos | -- | -- | Sequencing not successful |
| 1181 | Sibu | Neg | Pos | -- | -- | Sequencing not successful |
| 2042 | Sibu | Pos | Pos | 98.7% | Human CoV 229E |  |
| 2082 | Sibu | Pos | Neg | -- | -- |  |
| 2098 | Sibu | Neg | Pos | -- | -- | Sequencing not successful |
| 2099 | Sibu | Neg | Pos | -- | -- | Sequencing not successful |
| 2112 | Sibu | Neg | Pos | -- | -- | Sequencing not successful |
| 3003 | Kapit | Pos | Pos | 99.8% | Human CoV 229E |  |
| 3067 | Kapit | Pos | Neg | -- | -- |  |
| 3073 | Kapit | Pos | Neg | -- | -- |  |
| 3104 | Kapit | Neg | Pos | -- | -- | Sequencing not successful |
| 3105 | Kapit | Neg | Pos | -- | -- | Sequencing not successful |
| 3109 | Kapit | Neg | Pos | -- | -- | Sequencing not successful |
| 3123 | Kapit | Neg | Pos | -- | -- | Sequencing not successful |

Supplementary Table 6. Comparison of positivity of panspecies conventional PCR versus real-time PCR for AdV

|  | rPCR - | rPCR + | Total |
| --- | --- | --- | --- |
| Panspecies - | 489 | 32 | 521 |
| Panspecies +^*^ | 44 | 30 | 74 |
| Total | 533 | 62 | 595^†^ |

*Panspecies + indicates the panspecies conventional PCR assay yielded an amplicon of the expected molecular weight. We characterized 30 of these 74 Panspecies + viruses through sequence typing of that amplicon; ^†^excludes suspect-positive results.

Supplementary Table 7. Comparison of positivity of panspecies conventional PCR versus real-time PCR for EV

|  | rRT-PCR - | rRT-PCR + | Total |
| --- | --- | --- | --- |
| Panspecies - | 523 | 23 | 546 |
| Panspecies +^*^ | 46 | 2 | 48 |
| Total | 46 | 25 | 594^†^ |

*Panspecies + indicates the panspecies conventional RT-PCR assay yielded an amplicon of the expected molecular weight. We characterized 17 of these 48 Panspecies + viruses through sequence typing of that amplicon; ^†^excludes suspect-positive results.

Supplementary Table 8. Comparison of positivity of panspecies conventional PCR versus real-time PCR for CoV

|  | rRT-PCR - | rRT-PCR + | Total |
| --- | --- | --- | --- |
| Panspecies - | 547 | 4 | 551 |
| Panspecies +^*^ | 45 | 2 | 47 |
| Total | 592 | 6 | 598^†^ |

*Panspecies + indicates the panspecies conventional RT-PCR assay yielded an amplicon of the expected molecular weight. We characterized 3 of these 47 Panspecies + viruses through sequence typing of that amplicon; ^†^excludes suspect-positive results.

References

1. Jain, S., et al., *Community-Acquired Pneumonia Requiring Hospitalization among U.S. Children.* New England Journal of Medicine, 2015. **372**(9): p. 835-845.

2. Jain, S., et al., *Community-Acquired Pneumonia Requiring Hospitalization among U.S. Adults.* New England Journal of Medicine, 2015. **373**(5): p. 415-427.
